# Design of a Compact Dual-Band and Dual-Mode Wearable Antenna for WBAN Applications

**DOI:** 10.3390/s25113361

**Published:** 2025-05-27

**Authors:** Wei Zhang, Wenran Li, Xiaoyu Feng, Chen Zhao, Yan Li, Xiaoyi Liao

**Affiliations:** 1 School of Electronics and Information Engineering, Nanjing University of Information Science and Technology, Nanjing 210044, China; 202283270161@nuist.edu.cn (W.Z.); lwr9612@163.com (W.L.); liyan11@nuist.edu.cn (Y.L.); liaoxiaoyi2009@163.com (X.L.); 2 Key Laboratory of Radar Imaging and Microwave Photonics, Nanjing University of Aeronautics and Astronautics, Nanjing 210016, China; 3 School of Information and Communication Engineering, Dalian University of Technology, Dalian 116081, China; fxyuuu@163.com

**Keywords:** dual-band, dual-mode, wearable antenna, ISM, WBAN

## Abstract

This paper presents a novel design of a compact dual-band dual-mode wearable antenna. The antenna is fed through a single coaxial feed probe, which excites TM_01_ and TM_11_ modes at 2.45 GHz and 5.8 GHz, respectively. These modes exhibit distinct radiation characteristics. The omnidirectional TM_01_ mode at 2.45 GHz is suitable for on-body communication, while the directional TM_11_ mode at 5.8 GHz is more appropriate for off-body communication. The antenna prototype was fabricated and measured. The measured performance is consistent with the simulations. Additionally, further simulations and measurements were conducted to verify the interactions between the proposed antenna and the human body. The results demonstrate that the proposed antenna exhibits significant potential as a candidate for wireless body area network (WBAN) communications.

## 1. Introduction

Wireless body area networks (WBANs) are wireless systems that operate around the human body, enabling seamless wireless communication between body-worn sensors and external devices. In WBANs, two typical communication modes are often used, namely on-body communication and off-body communication [1]. In a WBAN system for on-body and off-body communications, it is essential to design antennas with specific operating frequencies and radiation patterns to accommodate the diverse communication requirements effectively. On the one hand, because of the constant movement of the human body, wearable antennas for on-body communication should exhibit omnidirectional radiation to facilitate communication across various body regions. On the other hand, off-body communication, which typically involves longer communication distances, benefits from broadside radiation patterns. Moreover, lower frequencies, characterized by longer wavelengths and stronger diffraction, are more suitable for on-body communication. Higher frequencies are preferred for off-body communication to achieve increased gain and reduced back lobe radiation. Further, for off-body communication, as the human body’s position changes significantly with movement, circular polarization (CP) is favored to mitigate polarization mismatch. Apart from the radiation characteristics, the interaction between the antenna and the human body is crucial for wearable antennas. Ideally, the human body should have minimal impact on the performance of the antenna, while the energy absorbed by the human body from the antenna should be kept at a safe level. Therefore, the specific absorption rate (SAR) should also be an important standard for measuring the performance of the antenna.

Dual-band wearable antennas have attracted significant attention for the application of on/off body communications. In [2], a wearable patch antenna operating at 2.45/5.8 GHz is proposed by cutting four L-shaped slots on the ground plane. CP is achieved at 5.8 GHz through the combination of the L-shaped slots and the patch antenna. In [3], an antenna of compact size operating at 2.45/5.8 GHz is achieved by cutting an open-ended quarter-wavelength slot on a quadrant circular patch antenna. In [4], a dual-band textile antenna has been proposed with AMC to reduce back radiation. Recently, in [5], a dual-band wearable antenna was analyzed and designed with the guidance of characteristic mode theory (CMT). However, all these designs exhibit either directional or omnidirectional radiation patterns at both operating frequencies, which is obviously not a good choice for on-body and off-body communications. In [6,7], dual-band wearable antennas with distinct radiation properties have been proposed for ISM frequency bands, achieving directional radiation at the lower frequency and omnidirectional radiation at the higher frequency. This discrepancy can lead to high propagation loss, which is not ideal for on/off body communication. Additionally, the slots on the ground plane contribute to the radiation patterns that are sensitive to the human body. While some wearable antennas have demonstrated satisfactory radiation characteristics for on/off body communications [8,9,10,11], these designs involve complex structures with multiple ports or switches, which limit the antenna’s flexibility and adaptability to different application scenarios. In [12,13], antennas with proper radiation characteristics at 2.45/5.8 GHz have been proposed using a single feed without the need for switches. However, both of these antennas incorporate an air gap, requiring multiple substrates with a high profile. Additionally, their SAR values are also relatively high.

To tackle these issues, a dual-band, dual-mode, dual-polarization antenna is proposed in this paper. The antenna incorporates a ring patch and four short pins to the traditional single-fed circularly polarized patch antenna, introducing an additional operating frequency of 2.45 GHz alongside the original 5.8 GHz frequency band, forming a dual-band antenna. Additionally, an arc-shaped slot is integrated into the ring patch to adjust the antenna’s maximum radiation direction at 5.8 GHz. The antenna not only features dual-band, dual-mode, and dual-polarization capabilities, demonstrating strong adaptability to a variety of communication scenarios, but it also features a simple structure configuration and compact size, enhancing its wearability. With all these features, the proposed antenna turns out to be a promising candidate for WBAN applications.

## 2. Antenna Design and Performance

### 2.1. Antenna Geometry

**Figure 1:**
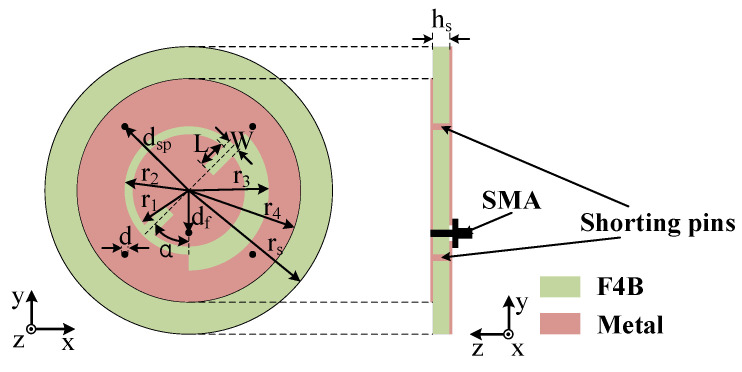
Configuration of the proposed antenna.

The configuration of the proposed antenna is shown in Figure 1. It is printed on an F4B substrate with *ε_r_* = 2.55 and *tanδ* = 0.002. As a common material in the production of high-frequency PCB boards, F4B has a relatively mature production process and low cost, which improves the cost performance of this antenna. The substrate thickness is denoted as *h_s_*. As is shown, the main radiator of the antenna consists of a central circular patch surrounded by a ring patch, both of which are printed on the top surface of the substrate. The role of a ring patch is to provide a new radiation pattern and polarization mode for the antenna [14,15,16]. The full ground plane is located on the bottom surface of the substrate, which helps suppress the back radiation of the human body [17] and reduce the influence of the distance between the antenna and the human body on the antenna performance [18,19]. A pair of symmetrical slots are cut from the two patches to achieve the circular polarization and radiation properties [20]. Four shorting pins are placed connecting the ring patch and the metallic ground plane to improve the impedance matching of the antenna at a lower frequency. The parameters are also labeled in Figure 1, and the antenna dimensions are as follows: *r*_1_ = 7.05 mm, *r*_2_ = 8.25 mm, *r*_3_ = 10.25 mm, *r*_4_ = 20.65 mm, *r_s_* = 28 mm, *d* = 1.6 mm, *d_sp_* = 14.9 mm, *d_f_* = 5.7 mm, *L* = 4.2 mm, *W* = 1.9 mm, *a* = 45°, and *h_s_* = 5 mm.

### 2.2. Design Process and Operating Mechanism

The proposed antenna utilizes two different radiation modes. The central circular patch supports the traditional cavity mode, providing broadside radiation at a higher frequency. To achieve directional circular polarization, two rectangular slots of dimensions of *L* × *W* are cut from the central circular patch. Meanwhile, the ring patch loaded with shorting pins produces an omnidirectional monopolar patch mode at a lower frequency. To ensure that the radiation direction of the main lobe aligns with the +z axis, a segment of the ring patch corresponding to a metal arc with an angle of 135° is removed to fix the shape of the radiation pattern.

**Figure 2:**
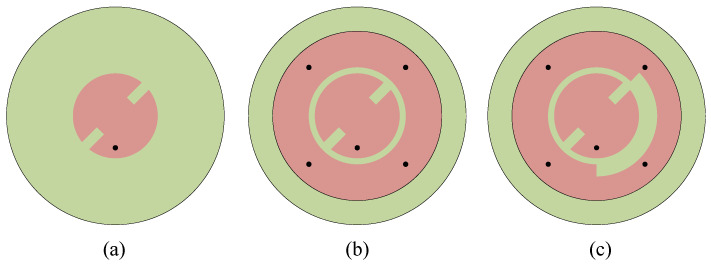
Design process of the proposed antenna: (**a**) Antenna 1, (**b**) Antenna 2, (**c**) Antenna 3.

Figure 2 illustrates how the proposed antenna evolves from the traditional CP patch antenna to the current configuration. Antenna 1 represents a conventional single-fed circular polarized patch antenna. In this design, two symmetrically small rectangles are cut from the circular patch, introducing a disturbance that is pivotal for the excitation of the two orthogonal modes, thereby enabling directional circular polarization.

**Figure 3:**
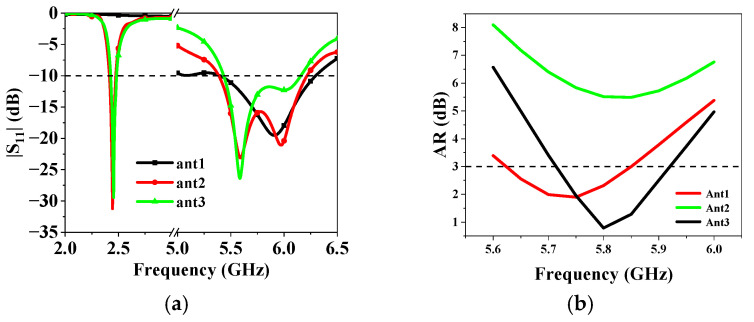
Simulated (**a**) |S_11_| and (**b**) AR for the antennas in Figure 2.

**Figure 4:**
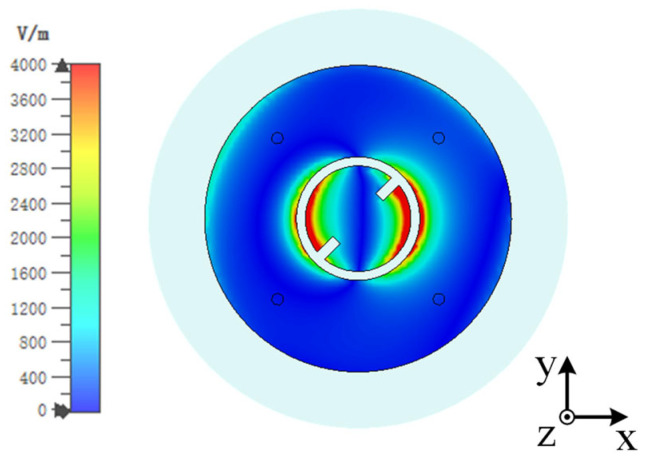
Simulated electric field distribution of Antenna 2 at 5.8 GHz.

Building upon Antenna 1, Antenna 2 incorporates a ring patch and four shorting pins around the periphery of the circular patch. The inner circular patch and the ring can be considered as a whole as a circular patch antenna whose fundamental mode is the TM_01_ mode, which supports an omnidirectional radiation pattern. The four shorting pins are applied to improve the impedance matching. Antenna 1 and Antenna 2 are simulated with CST Microwave Studio (MWS). The simulated |S_11_| parameters and AR are depicted in Figure 3, indicating that Antenna 1 operates at around 5.8 GHz. Antenna 2 maintains the high-frequency operation of Antenna 1 while introducing an additional resonance at 2.45 GHz. The AR of Antenna 1 is lower than 3 at 5.8 GHz, showing circular polarization properties. However, the AR of Antenna 2 deteriorated to about 5.5. This phenomenon could be explained by the field distribution of the antenna. Figure 4 illustrates the electric field distributions on the surface of the radiating patch at 5.8 GHz. Asymmetry of the feeding point results in an asymmetric electric field distribution across the gap between the inner circular patch and the ring patch, with more energy coupled to the bottom right that is stronger than that coupled to the orthogonal direction, increasing the AR of the antenna.

**Figure 5:**
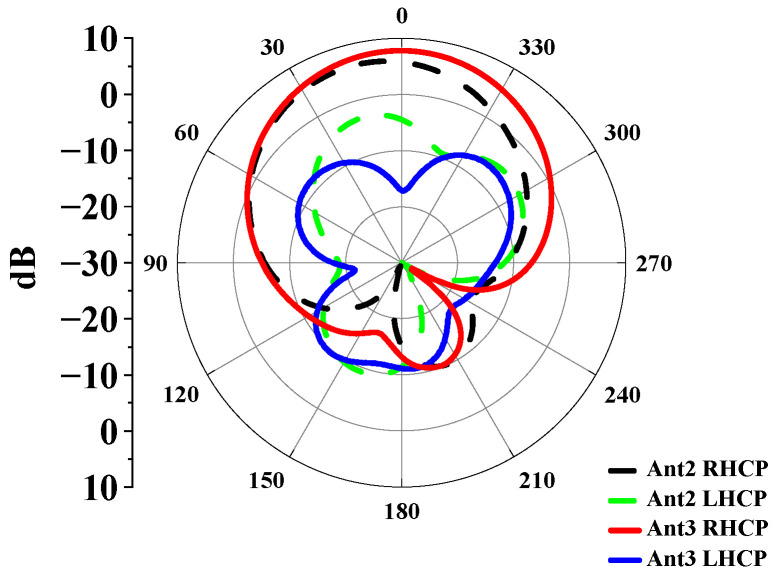
Simulated radiation patterns of Antenna 2 and Antenna 3 at 5.8 GHz.

The simulated radiation pattern of Antenna 2 at 5.8 GHz is presented in Figure 5. It can be noted that the maximum radiation direction deviates from the normal by approximately 15°. This can also be explained by the asymmetry of the electric field distribution. The electric field on the right-hand side is significantly stronger than that on the left, which adversely affects the main direction of the antenna’s radiation pattern.

**Figure 6:**
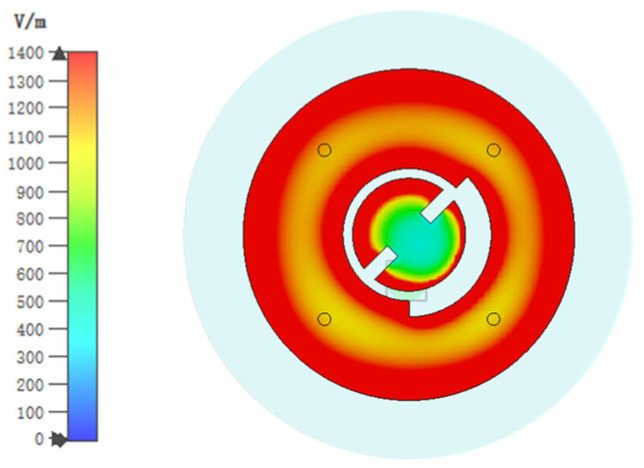
Simulated electric field distributions of Antenna 3 at 2.45 GHz.

To address this issue, Antenna 3 (the proposed antenna) was proposed. The coupling between the inner circular patch and the ring patch is reduced by removing an arc-shaped segment with a 135° central angle from the ring patch of Antenna 2. Antenna 3 is simulated with its S parameters and AR presented in Figure 3. As is seen, Antenna 3 exhibits almost the same impedance-matching bandwidth as Antenna 2, indicating that the modification did not adversely impact the antenna’s frequency response. More importantly, by removing an arc-shaped segment, the AR of the antenna is greatly improved. The radiation pattern of Antenna 3 on the xoz plane at 5.8 GHz is presented in Figure 5. It could be observed that Antenna 3 not only aligns the maximum gain direction towards the +z direction but also improves the antenna gain because of better AR. This modification has successfully addressed the initial asymmetry issue in the AR and radiation pattern. At this frequency, the electric field distribution conforms to the TM_11_ mode, showing broadside radiation. Figure 6 illustrates the electric field distributions on the surface of the radiating patch at 2.45 GHz. At this operating frequency, the field is predominantly concentrated on the metal ring, and the electric field beneath the patch points in the same direction. It can be clearly seen from this electric field that TM_01_ mode is excited, which supports omnidirectional radiation, making it well-suited for the application of on-body communications.

**Figure 7:**
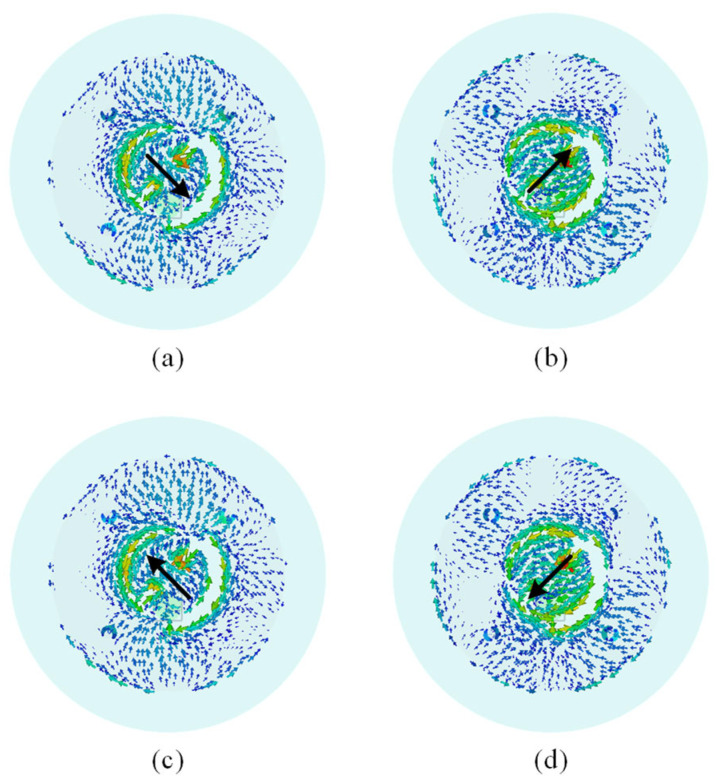
Simulated current distribution at 5.8 GHz: (**a**) t = 0, (**b**) t = T/4, (**c**) t = T/2, (**d**) t = 3T/4.

Figure 7 depicts the current distribution on the radiating patches at 5.8 GHz over one cycle. It can be seen that the current flows in a counterclockwise direction, corresponding to right-hand circular polarization (RHCP). This confirms that the antenna effectively produces the desired polarization at the higher frequency band.

**Figure 8:**
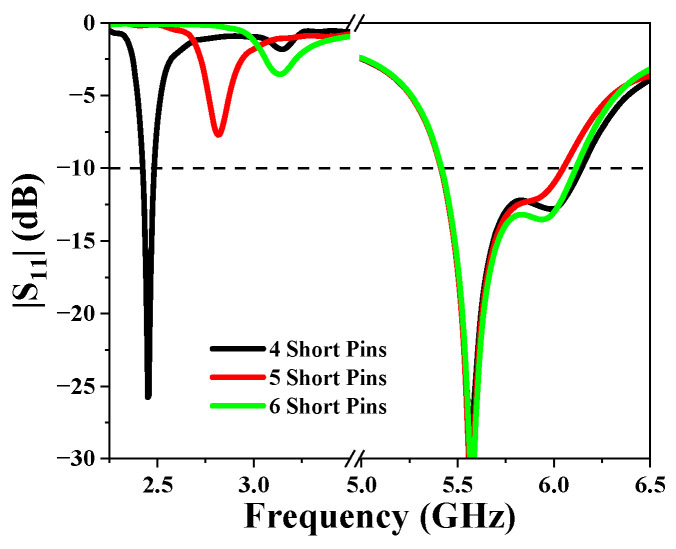
Simulated |S_11_| for different numbers of shorting pins.

The effect of some key parameters on the performance of the antenna is discussed. First of all, the effect of the number of shorting pins was analyzed. Figure 8 shows the |S_11_| of the antenna when the number of shorting pins increases from 4 to 6. It can be seen that the number of shorting pins mainly affects the resonant frequency and impedance matching of the antenna in the lower frequency band. As the number of shorting pins increases, the resonant frequency of the antenna in the low-frequency band gradually shifts to the high frequency. The selection of four short pins enables the antenna to operate at the desired frequency of approximately 2.45 GHz in the lower frequency band. Additionally, it ensures superior impedance matching for the antenna.

**Figure 9:**
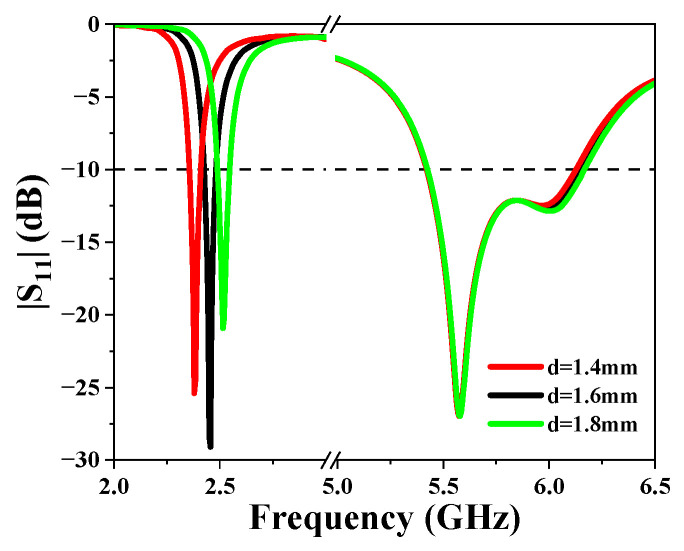
Simulated |S_11_| for different values of *d*.

Figure 9 shows how the diameter of the shorting vias *d* affects the resonant frequencies. As is shown, the diameter of the vias has a great influence on the lower resonant mode. When *d* increases, the resonant frequency of the first mode increases. This is because of two reasons. First of all, the inductance of the vias reduces with increasing *d*, leading to higher resonant frequency. Secondly, the resonant path is also reduced. On the other hand, the operating frequency of the higher operating band does not change. We set the value of *d* to 1.6 mm to ensure that the antenna operates at exactly 2.45 GHz.

**Figure 10:**
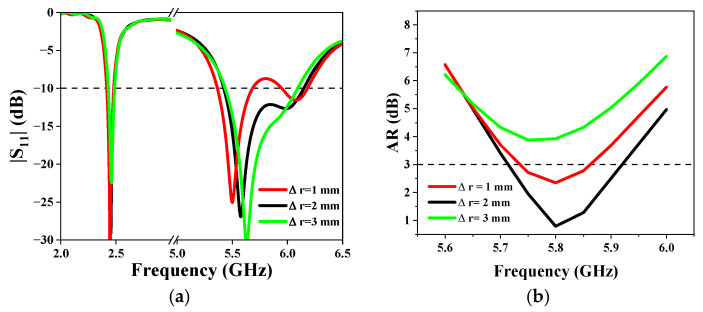
Simulated (**a**) |S_11_| and (**b**) AR for different values of Δr.

In addition, the size of the removed arc-shaped segment will also affect the performance of the antenna. Figure 10a presents the effect of the size of the arc (Δr = r_3_ − r_2_) on the S parameters of the antenna. As can be seen, the lower frequency almost does not change with the Δr. On the other hand, when Δr changes from 1 mm to 3 mm, the impedance-matching bandwidth does not change very much. Figure 10b shows the AR of the antenna. An optimal AR is obtained when Δr = 2 mm.

## 3. Antenna in Free Space

### 3.1. S-Parameter

**Figure 11:**
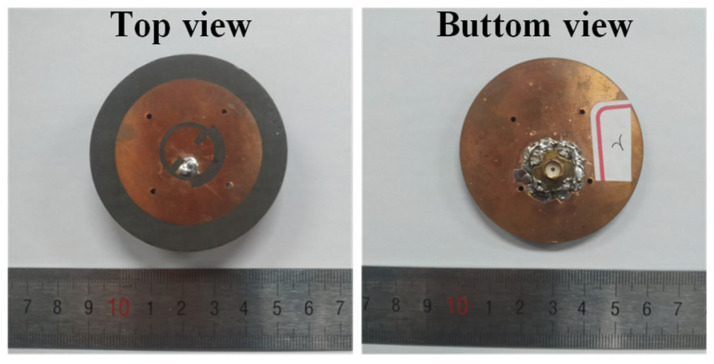
Photo of the fabricated antenna.

**Figure 12:**
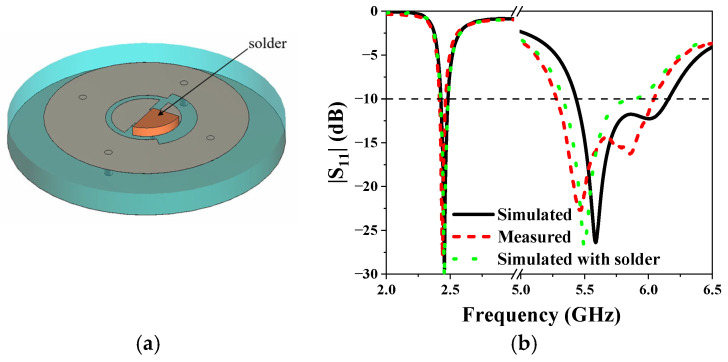
(**a**) Antenna model with solder. (**b**) Simulated and measured |S_11_| of the proposed antenna in free space.

The antenna was fabricated using the printed circuit technique to validate its simulated performance. The top and back views of the fabricated antenna are depicted in Figure 11. The fabricated antenna was measured with a vector network analyzer (VNA). Figure 12 presents both the measured and simulated S-parameters of the antenna. The measured |S_11_| closely aligns with the simulated data. A slight discrepancy at 5.8 GHz, where the measured operating frequency band is lower than the simulations, can be observed. This difference could be attributed to fabrication errors. A solder with a thickness of 2 mm is added at the feeding point in the antenna model (as shown in Figure 12a). The results show that the presence of solder does have a certain impact on the |S_11_| parameters of the antenna in the high-frequency band. With the effect of soldering, the measured |S_11_| matches better with simulations. At the lower frequency, the impedance bandwidth, defined for |S_11_| < −10 dB, ranges from 2.419 GHz to 2.461 GHz. In contrast, at the higher frequency, the impedance bandwidth is from 5.28 GHz to 6.04 GHz. These measurements confirm that the antenna could operate effectively across the specified dual-frequency bands.

### 3.2. Axial Ratio and Gain

**Figure 13:**
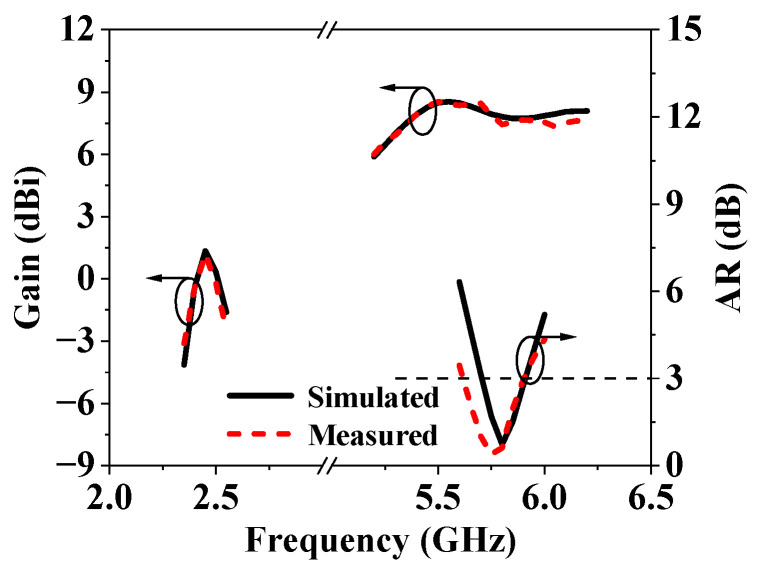
Simulated and measured gain and AR in free space.

The radiation properties of the fabricated antenna were measured in a compact range microwave anechoic chamber. The axial ratio (AR) and gain are presented in Figure 13. CP is realized with a measured 3 dB axial ratio bandwidth (ARBW) ranging from 5.62 GHz to 5.90 GHz, corresponding to a relative bandwidth of 4.8%. Additionally, the peak gain in the xoy plane is 1.20 dBi at 2.45 GHz, while at 5.8 GHz, the gain is measured at 7.45 dBi. The measured gains are a bit lower than those in the simulations (1.35 dBi and 7.81 dBi). Despite this minor discrepancy, the antenna demonstrates satisfactory performance in terms of both polarization and gain across the specified frequency bands.

### 3.3. Radiation Patterns

**Figure 14:**
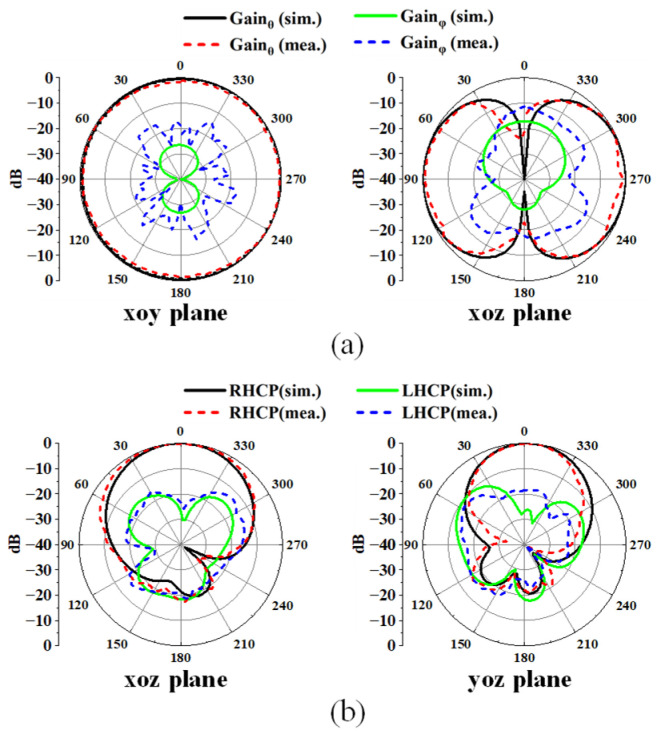
Simulated and measured radiation patterns in free space at (**a**) 2.45 GHz and (**b**) 5.8 GHz.

In Figure 14, the radiation patterns at the two operating frequencies from both simulation and measurement are compared, showing relatively close agreement. At 2.45 GHz, the antenna exhibits good omnidirectional characteristics in the xoy plane, with cross-polarization levels lower than −15 dB. This performance is well-suited for on-body communication applications, where minimizing cross-polarization is crucial to reduce interference. At 5.8 GHz, the antenna mainly radiates along the normal direction, with a front-to-back ratio exceeding 15 dB. This feature is advantageous for off-body communication, as it ensures a strong signal towards the intended direction while suppressing radiation in the opposite direction. Overall, the antenna’s radiation patterns at both frequencies meet the requirements for effective communication in both on-body and off-body scenarios.

### 3.4. The Performance of the Antenna Under Bending Conditions

**Figure 15:**
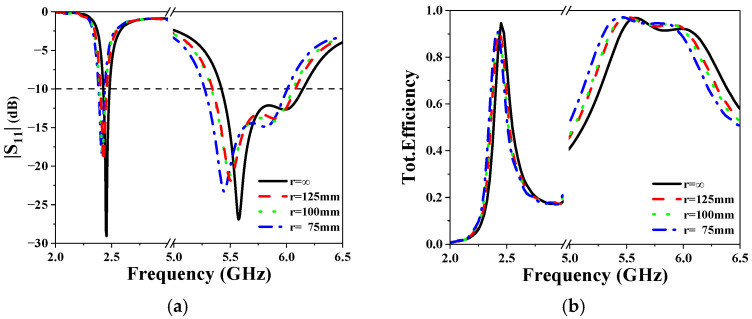
Simulated (**a**) |S_11_| and (**b**) efficiency under bending scenarios.

The performance of the antenna under bending conditions, an important indicator of wearable antennas [21,22,23], has also been simulated. We respectively simulated the |S_11_| parameters of the antennas with bending radii at 75 mm, 100 mm, and 125 mm. The results are presented in Figure 15. It can be observed that little frequency shift occurred under bending. In addition, the efficiency remains above 80% at lower operating frequencies and above 90% at higher frequencies. Overall, the antenna demonstrated excellent performance even when bent.

## 4. Antenna on the Human Body

### 4.1. Effect of the Human Body on the Antenna

**Figure 16:**
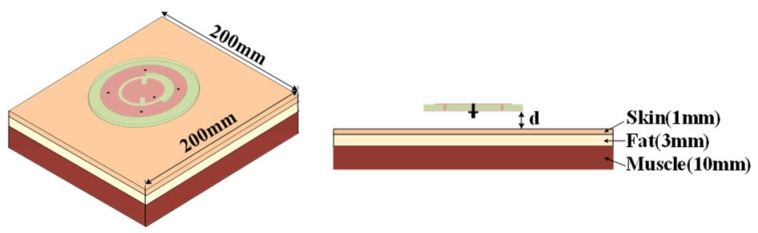
Configuration of the proposed antenna on the multilayer tissue.

**Figure 17:**
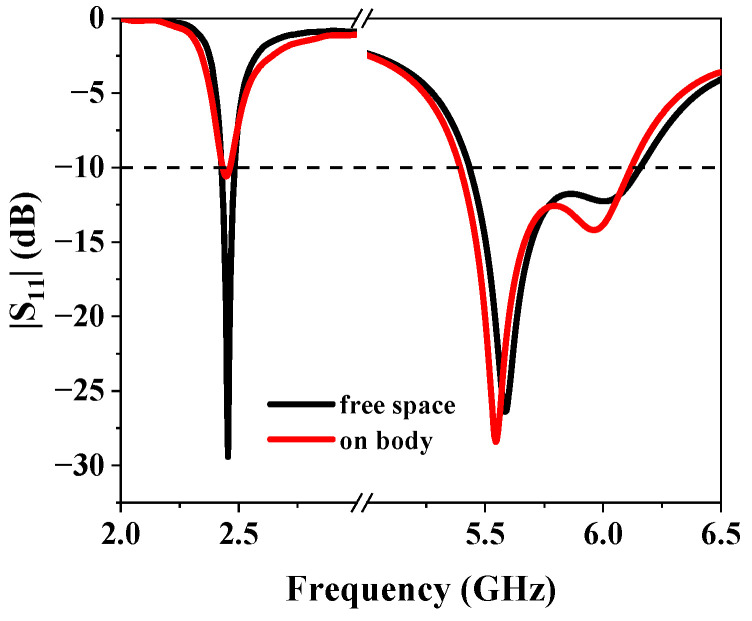
Simulated |S_11_| in free space and on body.

**Figure 18:**
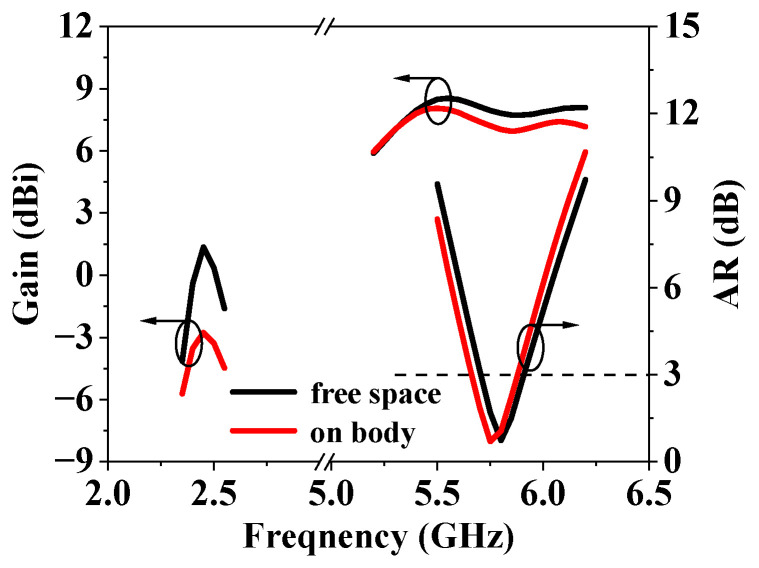
Simulated gain and AR in free space and on human body.

The human body can be considered as lossy high dielectric constant materials, which may affect the performance of the antenna. In this section, the three-layer human body tissue model (Figure 16) is established to study the influence of the human body on the proposed antenna. The parameters describing the properties of each tissue at 2.45/5.8 GHz are presented in Table 1. Figure 17 compares the simulated |S_11_| results of the proposed antenna when it is placed in free space and 5 mm above the human body model. From Figure 13, it can be seen that the antenna’s impedance bandwidth decreases from 55 MHz to 41 MHz at the lower operating band. On the other hand, the antenna maintains good impedance matching at both 2.45 GHz and 5.8 GHz. Obviously, the reflection coefficient of the antenna at lower frequency is more affected because of the omnidirectional radiation pattern. But it still could operate properly at the center frequency of 2.45 GHz. Figure 18 illustrates the AR and gain in free space and on human body. It should be noted that for lower frequency, the gain presented is for the xoy plane rather than the maximum gain. Similar to the result of S-parameter analysis, the AR and gain of the antenna at higher frequencies are less affected by the presence of human tissue. The gain at the lower frequency decreases due to strong diffraction, which further represents that the omnidirectional mode is more suitable for on-body communication.

**Figure 19:**
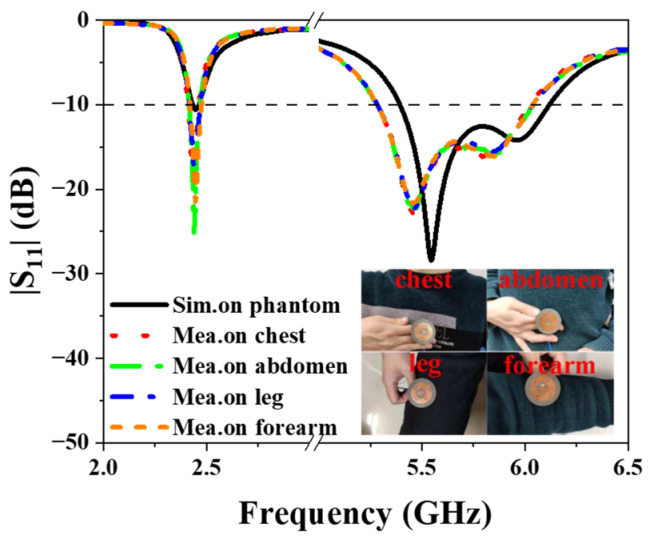
Simulated and measured |S_11_| at different positions on human body.

The |S_11_| is also measured when it is placed at different positions on human body, including the chest, the abdomen, the leg, and the forearm. The measurement setup and the measured results are presented in Figure 19. It can be observed that the human body has little impact on the antenna’s performance, which aligns with the simulations.

**Figure 20:**
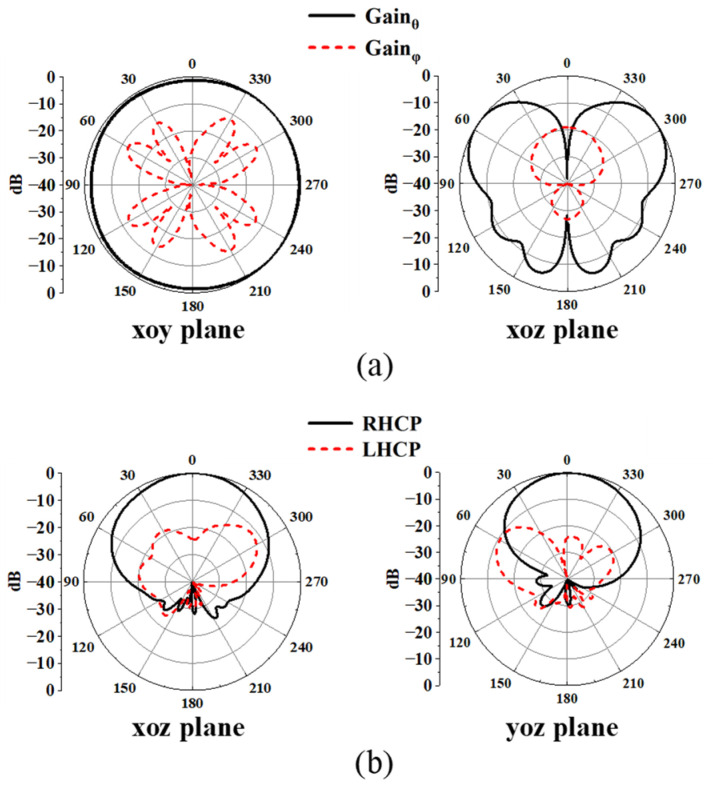
Simulated radiation patterns on human body at (**a**) 2.45 GHz and (**b**) 5.8 GHz.

Figure 20 displays the simulated radiation pattern of the proposed antenna when it is positioned 5 mm above the human body. At 2.45 GHz, the existence of the human body causes the main radiation direction to tilt upwards, leading to a reduction in gain on the xoy plane. Nonetheless, the antenna continues to exhibit a good omnidirectional radiation pattern. Concurrently, the radiation pattern at 5.8 GHz remains almost unchanged. Because of the absorption of microwave energy by the human body, a decrease in the back lobe can be observed.

### 4.2. SAR Analysis

**Figure 21:**
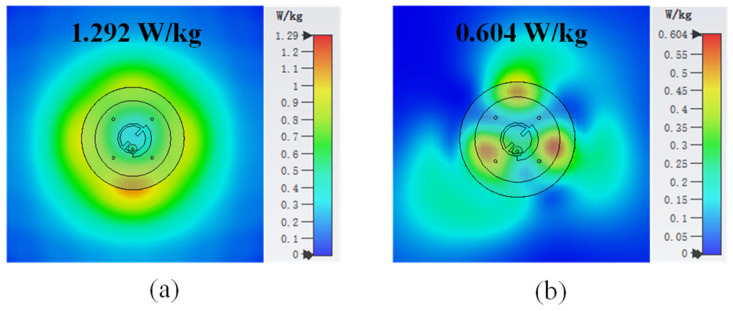
The 1-g SAR distribution on the multilayer tissue model at (**a**) 2.45 GHz and (**b**) 5.8 GHz.

To evaluate the safety of the designed antenna when operating in close proximity to the human body, the SAR of the antenna was studied using CST. The SAR estimation was conducted with the antenna positioned 5 mm above the aforementioned human tissue model, which measures 200 × 200 × 14 mm^3^. The resulting simulated SAR characteristics at an input power of 0.5 W are depicted in Figure 21. Under the 1-g standard, the SAR values at 2.45/5.8 GHz are 1.292 W/kg and 0.604 W/kg, which comply with the safety standard of 1.6 W/kg. Based on the SAR limit specified in the standard, the maximum allowable input power is 0.62 W, which is sufficient to support the applications of WBAN communication.

Finally, the antenna is compared with other published dual-frequency antennas, as shown in Table 2. It can be seen that compared with other antennas, although the size of the proposed antenna is a bit larger, it has a larger bandwidth and gain in the high-frequency band and also maintains good performance in the low-frequency band. In addition, the proposed antenna supports omnidirectional radiation at lower frequencies and circularly polarized broadside radiation at higher frequencies, which meet the requirements of on/off-body communications. To reduce the size of the antenna, potential approaches such as using a dielectric substrate with a higher dielectric constant or introducing extra capacitive or inductive loadings can be employed.

## 5. Conclusions

In this paper, a novel design of a dual-band, dual-mode, and dual-polarized wearable antenna has been proposed and studied. The antenna structure features a compact and simple structure. An additional omnidirectional TM_01_ mode is introduced by incorporating an annular metal structure with four shorting pins. Additionally, by adjusting the shape of the ring structure, the direction of the main beam is corrected. Both simulation and measurement have been carried out to demonstrate that the antenna could operate effectively in the presence of the human body and conform to the SAR value standards.

## Figures and Tables

**Table 1:** Tissue characteristics.

Tissue	2.45 GHz		5.8 GHz		Thickness (mm)
	ε_r_	tan δ	ε_r_	tan δ	
Skin	38.007	0.28262	35.114	0.32807	1
Fat	5.2801	0.14524	4.9549	0.18335	3
Muscle	52.729	0.24194	48.485	0.31715	10

**Table 2:** Performance comparison for the proposed antenna with other reference wearable antennas.

Ref.	H (mm) × Area (mm^2^)	f1 and f2 (GHz)	BW (%)	Gain (dBi)	Polarization	Rad.Pat.
[2]	5 × 1963	2.45/5.8	10/9	−5.1/3.3	L/C	O/O
[3]	1.58 × 986	2.45/5.8	1.2/2.0	2.13/5.16	L/L	B/B
[6]	1.6 × 1200	2.45/5.8	2.04/3.44	5.08/6.33	N	B/O
[11]	~1.58 × 9503	2.45/5.8	3.54/3.45	0.75/5.4	L/L	O/B
[12]	9.8 × 1018	2.45/5.8	N	−0.6/4.3	L/L	O/B
Proposed	5 × 2463	2.45/5.8	1.71/13.10	1.2/7.45	L/C	O/B

L: linearly polarized, C: circularly polarized, O: omnidirectional, B: broadside, N: not mentioned.

## Data Availability

Data are contained within the article.
